# Increased Risk of Death Triggered by Domestic Violence, Hunger, Suicide, Exhausted Health System during COVID-19 Pandemic: Why, How and Solutions

**DOI:** 10.3389/fsoc.2021.648395

**Published:** 2021-06-08

**Authors:** Kenneth Bitrus David, Abdullahi Tunde Aborode, Damilola Quazeem Olaoye, Ndikpongkeabasi Victor Enang, Aboaba Kazeem Oriyomi, Ismaeel Yunusa

**Affiliations:** ^1^ Hull York Medical School, University of Hull, Heslington, United Kingdom; ^2^ Faculty of Pharmaceutical Sciences, Kaduna State University, Kaduna, Nigeria; ^3^ Department of Chemistry, University of Ilorin, Ilorin, Nigeria; ^4^ Faculty of Pharmacy, University of Ibadan, Oyo, Nigeria; ^5^ Faculty of Pharmacy, University of Uyo, Uyo, Nigeria; ^6^ Department of Agricultural Economics, Federal University of Agriculture, Abeokuta, Nigeria; ^7^ Department of Clinical Pharmacy and Outcomes Sciences, University of South Carolina College of Pharmacy, Columbia, SC, United States

**Keywords:** suicide, pandemic, hunger, death, COVID-19, SARS-CoV-2, domestic-violence

## Abstract

Severe acute respiratory syndrome coronavirus 2 (SARS-CoV-2) infections, just like many other public health emergencies, is a well-established global health burden that has resulted in several changes in routines and lifestyles of people globally. The coronavirus disease (COVID-19) pandemic, caused by SARS-CoV-2, has directly or indirectly involved in the loss of lives of more than 3.24 million as of 6th May, 2021. The increasing threats posed by this pandemic were subsided by the swift and drastic measures put in place by different countries. As other causes of death before the emergence of COVID-19 still exist, the pandemic has further worsened their impact. The increased risks of COVID-19 deaths are not only due to the health burden it possesses, but also due to some other factors. These factors include domestic violence that becomes rampant, especially during lockdowns; hunger due to low economic development, unemployment, and loss of jobs; suicide due to depression; exhausted health system due to high level of COVID-19 cases and inability to contain it. As we move from the response phase into recovery, the pandemic’s direct and broader impacts on individuals, households, and communities will influence the capacity to recover. An understanding of these impacts is therefore required to develop priorities to support recovery. This paper identifies other causes of death amidst the pandemic, such as domestic violence, hunger, suicide, and exhausted health system, and how to minimize their effects.

## Introduction

The current COVID-19 pandemic is one of the largest respiratory disease outbreaks affecting several countries simultaneously and has affected over 200 countries as of April 2021 (David and Ozuluoha, 2020). As we move from the response phase into recovery, the pandemic’s direct and broader impacts on individuals, households, and communities will influence the capacity to recover. An understanding of these impacts is therefore required to develop priorities to support recovery (Bernard et al., 2020).

COVID-19 pandemic has affected many homes and families. It has caused numerous deaths in the world with greater collateral damages in some low and middle-income countries where people do not have access to proper healthcare, food, and jobs (Lim et al., 2020; Wang et al., 2020). These factors have, in turn, influenced other triggers of deaths during the COVID-19 pandemic. The numerous deaths triggered by COVID-19 and other factors that made the pandemic out of control, such as domestic violence, hunger, suicide, and exhausted health system, are discussed extensively in this paper, and suitable solutions that could reduce these risks are proposed in detail.

### Impacts of COVID-19 Pandemic on Human Lives

While different countries’ policies vary, a lockdown declaration seemed like a common occurrence across many countries (De Ver Dye et al., 2020). The declared lockdown, though having many advantages, came in with other notable disadvantages. Some of the considerable impacts are seen on the health of people with chronic diseases (Umaru et al., 2020; Aborode et al., 2020a), treatment of malaria (Aborode et al., 2021a), education (Lawal et al., 2020; Aborode et al., 2020b), drugs supply (Dada et al., 2020), healthcare services (David and Adebisi, 2020; Ahmad et al., 2021). The magnitude of these impacts has created an atmosphere where people are more susceptible to deaths not only directly linked to COVID-19 pandemic, but indirectly to other factors.

### Domestic Violence

COVID-19 has radically changed the lives of many individuals. During the quarantine mandated by governments to curb the spread of COVID-19, domestic violence made some people’s homes a more dangerous place to live and survive. This could be because they must stay the whole day with partners and away from people who can witness their experiences and give help. Many abusive situations were on an increase due to economic crises linked to COVID-19 emergence (many victims have difficulty leaving abusive partners for financial reasons). In addition, lockdown triggered the increased risk of domestic violence where parents fight with one another, children unable to handle the mental stress of such acts, thus increasing the prevalence of suicide, mental instability, and physical deformation (Elbert et al., 2018; Cluver et al., 2020). A recent study has proven a rise in domestic violence and social decadences during pandemics (Brooks et al., 2020). The rise in domestic violence during pandemics increases the level of violence and social menace that cannot be control easily due to low engagement of people and lockdown. Domestic violence during pandemics is linked to the adoption of negative stress coping mechanisms that occur between spouses, parents, and individuals during pandemic lockdowns. Lockdown also brings abusers closer to their victims for an extended period, consequently increasing the probabilities of repeated occurrences (Brenner, 1987; Reynolds et al., 2008; Palermo and Peterman, 2011).

Several media reports indicate a surge in domestic violence cases in various countries (Lima et al., 2020; van Gelder et al., 2020; Gearin and Knight, 2020). According to Kagi (2020), the overall crime rates in Australia have declined, however, the rate of domestic abuse increased by 5%. Some charities in Australia also raised concerns about COVID-19 misinformation used by the offenders to further control and abuse domestic violence victims (Kagi, 2020). Allen-Ebrahimian (2020) reported that China witnessed a threefold increase in domestic violence cases after imposing lockdown. Different states in the United States also reported about 21–35% increase in the number of domestic violence recorded (Wagers, 2020). According to Bradbury-Jones and Isham (2020), the lockdown imposed to deal with COVID-19 pandemic has granted greater freedom to abusers. It has become easier for the abusers to enforce control tactics by limiting the victims’ access to phones, the internet, and other people, while van Gelder et al. (2020) also emphasized that the lockdown limits familiar support options.

The proposed solutions to the occurrences of domestic violence are strengthening online support and aids systems (Haneef and Kalyanpur, 2020). There should be advocacy platforms aimed at curtailing the several factors catalyzing the spikes in violence against persons (WHO, 2020). Also, there should be proposed plans that will reduce the risk triggers of domestic violence, which results to death during COVID-19 pandemic. It is required that prioritizing violence prevention within the global public health agenda be encouraged. This can be done by defining the problem through the systematic collection of information, using research evidence to determine the causes and risk factors of violence, and implement effective interventions to prevent violence. Achieving these goals becomes particularly important during the pandemic because violence against women has dramatically increased (WHO, 2020). The effectiveness of online safety and health interventions for different needs of women who have experienced intimate partner violence have already been outlined previously (El-Serag and Thurston, 2020; Ford-Gilboe et al., 2020).

Moreover, to reduce the mortality rates due to domestic violence during COVID-19 pandemic, there is a need for funding sources to enhance telephone or remote counseling services with high-speed internet, hotlines, and emergence shelters. It is also mandatory to identify high-risk individuals and admonish them to avoid extreme events such as impulsive acts, homicide, or suicide.

### Hunger

Agriculture has remained the primary source of food globally, and hence agriculture plays a vital role in the nation’s economic development. The lockdowns imposed by countries have resulted in an obstruction to the free flow of all the stages involved in agriculture i.e. from farm to fork (including production, processing, distribution, and consumption). This has consequently resulted in a hike in the prices of food commodities (Torero, 2020). As a result of this increase in food prices and a further anticipated hike, food shortages, malnutrition, and even deaths have been recorded (Torero, 2020). The United Nations World Food Program has estimated that by the end of the year 2020, over 265 million people could suffer from food shortages and hunger (Food Security Information Network, 2020).

In a national survey conducted in the United States of America, it was shown that the COVID-19 pandemic has directly increased the rate of food insecurity in households having children (Bauer, 2020). This survey showed that 34.5% of households with a child  ≤18 years old and 34.4% of families with children  ≤12 years old were experiencing food shortages by the end of April 2020, compared with 14.7 and 15.1% in 2018, respectively (Bauer, 2020). It was also shown that 17.4% of mothers with children ≤12 years old reported that “the children in my household were not eating enough because we just couldn’t afford enough food,” compared to 3.1% in 2018 (Bauer, 2020).

COVID-19 pandemic aggravated the hunger crisis in the world’s hunger hotspots and created new epicenters of hunger worldwide. By the end of the year, 12,000 people per day died from COVID-19 pandemic, which is linked to hunger, potentially more than the disease (Siguerva et al., 2020). The pandemic is the final straw for millions of people already struggling with the impacts of conflict, climate change, inequality, and a broken food system that has impoverished millions of food producers and workers. During the COVID-19 pandemic, despite the socioeconomic disparities across borders and communities, there has been expansive togetherness, love, and care. The lockdown, however, introduced several economic hardships Siguerva et al. (2020); Aborode et al. (2021b), such as coronavirus famine, food insecurity and adverse hunger, which triggered risk of human death. Neglecting to combat hunger may have caused severe malnutrition and starvation, as evident in war-torn or ravaged ambient. These correlated to salient risk factors or determinants Chukwuma (2020) for compromised immune systems and facilitated susceptibility to infection rates, life-threatening disorders associated with the novel coronavirus. These disorders include severe respiratory distress, pneumonia, diarrhea, cholera, other gastrointestinal diseases Chaolin et al. (2020), and emerging and re-emerging diseases Chukwuma (2018) to poor sanitation and inadequate water supply.

To mitigate the impact of hunger during COVID-19 pandemic on human lives, there is a need for COVID-19 economic recovery palliative. This will provide families with prompt and adequate access to food and other resources during and after the pandemic crisis. There is also a need for an improvement in the nutritional intake of people who are vulnerable- mostly people with low standard of living and other factors that trigger their vulnerability such as health issues (El Zowalaty et al., 2020). Sustainable development policies, actions, and good governance will reduce and eventually eradicate the burden of poverty triggered by hunger (Chukwuma, 2020; Abdullahi et al., 2021). Sustainable steps need to be taken to prevent deaths secondary to hunger amidst the pandemic, as they can affect other sectors if not duly attended to because human survival depends on quality foods. In addition, there should be an assurance that food will be readily available to people who need them. This is to mitigate the consequences posed by hunger, which can result in death if there is no action implemented.

### Suicide

The potential of COVID-19 pandemic to cause long-lasting morbidity implies that it may serve as a risk factor for mental illness and suicide in the end. It was found that psychosis (a risk factor for suicide) was high in people during the H1N1, MERS, and SARS pandemics (Rogers et al., 2020). Wasserman (1992) stated that the Spanish flu epidemic (1918–1920) resulted in a slight increase in the number of suicide cases in the United States of America. In Hong Kong, the 2003 SARS epidemic also increased the rates of suicide cases (Cheung et al., 2008). Generally, few studies have investigated the impact of prior pandemics on suicide rates (Wasserman, 1992; Cheung et al., 2008).

The factors that result in suicide during COVID-19 are also due to the economic hardship faced by people, with loss of jobs occupying the premium position. Other factors that trigger suicides during the COVID-19 pandemic are entrapment, social isolation, alcohol consumption, and loneliness (O’Connor and Kirtley, 2018; David et al., 2020b).

To reduce the rate of suicide triggered by the COVID-19 pandemic, there should be programs and awareness campaigns organized by NGOs and government bodies that will ensure proper mental health education for people. These programs would inform them on how to take care of their mental health and why they need to take care of their loved ones. The lockdowns imposed in several parts of the world have resulted in economic hardship and loss of jobs for many people, thus creating financial stressors (Cheung et al., 2008). These are among the risk factors for suicide. The government should provide financial security to ease the hardship posed by these circumstances. These can be in the form of housing, food, employment support, and consideration for their future and not just their current situations. There should be a responsible reporting of suicide cases so that people can maintain their emotional and mental stability; irresponsible reporting of spikes of suicide recorded instilled fear in people’s minds (Niederkrotenthaler et al., 2020). Finally, support in form of easy-accessible and well-distributed telephone help-lines should be available to help people with their mental health.

### Deaths Triggered by Exhausted Health System

The disruption of healthcare services to other medical conditions due to the drifted attention given to COVID-19 pandemic has resulted in many deaths (Santoli et al., 2020). The deaths triggered by healthcare inability to provide services to all patients was because of the priorities given to COVID-19 patients and suspected patients at the expense of others (Lange et al., 2020). During the pandemic, regular healthcare services inevitably reduced because of concerns about SARS-CoV-2 exposure, restructuring regular hospital facilities to facilitate the COVID-19 patients, shifting of health professionals from their professional department to COVID-19 departments, shortage of beds, shortage of operation theaters, and shortage of doctors and nurses (Babatunde et al., 2020; Aborode et al., 2021c). This has also decreased in-person services, and supported the use telehealth in order to address some healthcare needs of patients (Lange et al., 2020).

To solve the problem of increased deaths from other causes due to healthcare system’s inability to provide services to all patients, balancing the direct response to COVID-19 with the need for other health services’ continuous delivery is a universal dilemma for policymakers (Aborode et al., 2021d). It is particularly challenging for decision-makers in low- and middle-income countries (LMICs), where health systems already face enormous demands to address infectious and non-communicable diseases coupled with significant resource constraints (Oseran et al., 2020; Abdullahi et al., 2020). The leaders should evaluate different policy options that will effectively respond to COVID-19 by exacerbating all the causes of morbidity and mortality among the population from neglecting or diverting care for other conditions (Stuckler et al., 2009).

Furthermore, exploring underlying reasons for medical care avoidance should be act on, which include people with disabilities, people with underlying health conditions, unpaid caregivers for adults, and those who face structural inequities. If care for population survival rate and standard of living were neglected because of concerns about SARS-CoV-2 exposure or if there were closures or limited options for in-person services, providing accessible telehealth or in-home health care could address some of these issues (Oseran et al., 2020).

Communities, health care systems, and public health agencies should foster equity by working together to ensure access to information, testing, and care to all. The higher prevalence of medical care delay or avoidance among patients with high medical service bills or payments on people with no money might reflect differences in medical care-seeking behaviors (Chan et al., 2020).

## Conclusion

The spread of the COVID-19 pandemic has resulted in many unprecedented events and an increase in the number of non-COVID-19 deaths. To deal with the direct effects of the pandemic and prior to universally available vaccination, many governments have imposed lockdowns to reduce the viral spread. This, however, has resulted, in social distancing, economic instability, mental health problems, isolation, depression, domestic violence, suicide, hunger, and a strained healthcare system that was not able to provide services as usual (David et al., 2020a). Although there have been studies exploring the impact of the COVID-19 pandemic, analyzing the causes of death triggered by other factors such as domestic violence, suicide, hunger, and exhausted healthcare system is of great importance. There is a need for NGOs, governments, and individuals to play a role in mitigating these challenges.

## Data Availability

There is no data available for this study as this is a perspective research study..
